# Effect of Antiepileptic Drugs for Acute and Chronic Seizures in Children with Encephalitis

**DOI:** 10.1371/journal.pone.0139974

**Published:** 2015-10-07

**Authors:** Kuang-Lin Lin, Jainn-Jim Lin, Shao-Hsuan Hsia, Min-Liang Chou, Po-Cheng Hung, Huei-Shyong Wang, CHEESE Study Group

**Affiliations:** 1 Division of Pediatric Neurology, Chang Gung Children’s Hospital and Chang Gung Memorial Hospital, Chang Gung University College of Medicine, Taoyuan, Taiwan; 2 Chang Gung Children’s Hospital Study Group for Children with Encephalitis/Encephalopathy Related Status Epilepticus and Epilepsy (CHEESE), Taoyuan, Taiwan; 3 Division of Pediatric Critical Care and Emergency Medicine, Chang Gung Children’s Hospital and Chang Gung Memorial Hospital, Chang Gung University College of Medicine, Taoyuan, Taiwan; 4 Graduate Institute of Clinical Medical Sciences, Chang Gung University College of Medicine, Taoyuan, Taiwan; University of Modena and Reggio Emilia, ITALY

## Abstract

**Background:**

Encephalitis presents with seizures in the acute phase and increases the risk of late unprovoked seizures and epilepsy. This study aimed to evaluate the effect of antiepileptic drugs in pediatric patients with acute seizures due to encephalitis and epilepsy.

**Patients and Methods:**

Cases of acute pediatric encephalitis between January 2000 and December 2010 were reviewed. Clinical data, including onset at age, seizure type, seizure frequency, effects of antiepileptic drugs, and prognosis were analyzed.

**Results:**

During the study period, 1038 patients (450 girls, 588 boys) were enrolled. Among them, 44.6% (463) had seizures in the acute phase, 33% had status epilepticus, and 26% (251) developed postencephalitic epilepsy. At one year of follow-up, 205 of the 251 patients with postencephalitic epilepsy were receiving antiepileptic drugs while 18% were seizure free even after discontinuing the antiepileptic drugs. Among those with postencephalitic epilepsy, 67% had favorable outcomes and were using <2 anti-epileptic drugs while 15% had intractable seizures and were using ≥ 2 antiepileptic drugs. After benzodiazepines, intravenous phenobarbital was preferred over phenytoin as treatment of postencephalitic seizures in the acute phase. For refractory status epilepticus, high-dose topiramate combined with intravenous high-dose phenobarbital or high-dose lidocaine had less side effects.

**Conclusions:**

Children with encephalitis have a high rate of postencephalitic epilepsy. Phenobarbital and clonazepam are the most common drugs used, alone or in combination, for postencephalitic epilepsy.

## Introduction

Encephalitis is a common central nervous system disorder in children. It refers to inflammation and swelling of the brain often caused by either a direct viral infection or an immune-mediated process. It is an important cause of acute symptomatic seizures and subsequent epilepsy [**1**–3]. Previous epidemiologic studies have shown that 2.7–27% of epilepsies are secondary to previous central nervous system infections [4–7]. Encephalitis-related seizures may present as a single seizure or as refractory status epilepticus, or even intractable epilepsy. But few studies have reported on the efficacy and choice of antiepileptic drugs for acute seizures, chronic seizures and epilepsy in children.

Acute central nervous system infections are most often evidenced in the first episode of status epilepticus and appear to be markers for morbidity and mortality [8]. Based on the underlying risk factors, optimal treatment should be given to minimize brain damage in the acute phase. If management is not initiated promptly, refractory status epilepticus may develop, with subsequent progression to intractable epilepsy. Postencephalitic epilepsy has been reported to become intractable in 40–50% of children [9,10]. This study, therefore, aimed to report clinical experience on the use of antiepileptic drugs in the management of acute and chronic seizures in children with encephalitis. The outcomes were analyzed after at least one year of follow-up.

## Materials and Methods

### Data source

All case records from the Department of Pediatrics, Chang Gung Children’s Hospital coded with the discharge diagnosis of acute encephalitis from January 2000 to December 2010 were reviewed. Encephalopathy was defined as at least one symptom or sign of parenchymatous brain dysfunction such as altered consciousness, personality or behavioral change, seizure, paresis, or ataxia. Encephalitis was defined as the presence of encephalopathy plus at least two of the following: (1) body temperature >38°C; (2) cerebrospinal fluid examination showing increased protein content >40 mg/dL and/or pleocytosis >5 white blood cells/uL; (3) abnormal electroencephalography (EEG) findings, such as diffuse or focal slow activity, or periodic lateralized epileptiform discharge; and (4) abnormal neuroimaging results including computed tomography (CT) and magnetic resonance imaging (MRI) [10–13]. Acute symptomatic seizures in this study were defined as seizures occurring within 7 days (acute phase) after onset of symptoms or signs of encephalitis. Unprovoked seizures were defined as seizures occurring in the absence of a potentially responsible clinical condition or beyond the interval estimated for the occurrence of acute symptomatic seizures [14]. The definition of postencephalitic epilepsy was modified as two or more unprovoked seizures after acute phase of encephalitis or recurrent seizures with abnormal EEG findings and/or MRI findings (high recurrence risk) at follow-up that required antiepileptic drugs more than 6 months after encephalitis [15–17].

### Patients

All of the children were previously healthy and none had prior seizures including febrile seizures. The exclusion criteria were age >18 years, purulent meningitis, prior neurological insult, progressive neurologic disorder, seizures due to electrolyte imbalance, or hypoglycemia. The Chang Gung Memorial Hospital’s Institutional Review Board approved this study (103-7263B). The need for informed consent was waived by the Institutional Review Board because the study was an observational, retrospective study using a database from which the patients’ identification information had been removed.

### Data collection

By chart review, data were collected, including age at onset, sex, presence or absence of seizures during the acute phase, initial seizure type, frequency of seizures (single, repetitive, status epilepticus, and refractory status epilepticus), response to antiepileptic drugs, mortality, and seizure outcomes of epilepsy. Repetitive seizures were defined as ≥2 seizures within the period of the acute phase, with the interval of 2 seizures >30 min. Status epilepticus was defined as continuous seizure activity lasting 30 min, or two or more discrete seizures between which consciousness was not fully regained [18]. Refractory status epilepticus was defined as seizures lasting more than 2 h despite treatment with conventional antiepileptic drugs.

Patients with status epilepticus were treated with a protocol that included initial therapy with a benzodiazepine, followed by therapeutic doses of phenytoin, phenobarbital, and/or valproic acid. If refractory status epilepticus was evident, the goal of treatment was to achieve complete clinical seizure control or a burst suppression pattern on electroencephalography. Treatment included multiple high-dose suppressive therapy of valproic acid, midazolam, propofol, thiopental, lidocaine, and high-dose phenobarbital [19]. The number of patients responding to the first, second, third, and fourth antiepileptic drugs was recorded. For a single seizure, repetitive seizures, or status epilepticus, the clinical cessation of convulsions was considered to be a therapeutic response. Mortality during the hospital stay and the causes were recorded.

Seizure outcomes were assessed on the last clinical visit. All patients with postencephalitic epilepsy with abnormal electroencephalography findings or recurrence of unprovoked seizures were treated with antiepileptic drugs for ≥6 months. The patients were then classified into three groups based on seizure outcomes after treatment at one year of follow-up: (1) intractable postencephalitic epilepsy; (2) favorable outcome; and (3) successful antiepileptic drugs withdrawal after 1 year of treatment. Intractable postencephalitic epilepsy was defined as more than two seizures per month in patients receiving two or more antiepileptic drug treatments. Favorable outcome was defined as either seizure-free or fewer than two seizure episodes per month after treatment. The follow-up period for the study group ranged from 1 year to 12 years and 5 months (mean±standard deviation (SD), 4.82±3.72 years).

### Statistical analysis

Statistical analysis was performed using the SPSS statistical software, version 12.0 (SPSS, Inc., Chicago, IL). Independent *t* test or one-way analysis of variance was used for continuous variables, and the χ2 or Fisher’s exact test was used for categorical variables. Statistical significance was set at *p*<0.05. All statistical tests were two-tailed.

## Results

### Demographic data

During the study period, 1138 patients were discharged with a diagnosis of acute encephalitis. After excluding 100 patients with underlying neurologic diseases, 1038 previously healthy patients diagnosed with acute encephalitis were enrolled in the study. There were 450 (43.4%) girls and 588 (56.6%) boys. Their mean age at onset of acute encephalitis was 6.07±4.47 years (range, 2 months to 18 years). Among them, 469 (45.2%) patients were aged ≤4 years, 288 (27.7%) were 5–8 years old, 163 (15.7%) were 9–12 years old, and 118 (11.4%) were 13–17 years old. The highest incidence of encephalitis was in the group aged ≤4 years. Most patients (n = 759, 72.9%) had encephalitis before the age of 8 years. The presumed infectious pathogens of children with acute seizures were showed in Table 1.

**Table 1:** The infectious pathogens of 463 children with different seizure frequency in acute phase.

	presumed infectious pathogens (*n*)					
Seizure frequency in acute phase	M. pneumonia	enterovirus	HSV	influenza viruses	VZV	unknown
Single seizure	13	3	11	12	7	37
Repetitive seizures	26	27	10	4	0	160
Status epilepticus	11	2	15	3	1	68
Refractory status epilepticus	11	0	5	2	0	35
Total (*n*)	61	32	41	21	8	300

M. pneumonia: mycoplasma pneumonia; HSV: herpes simplex virus; VZV: varicella-zoster virus

### Seizure profiles

During the acute phase of encephalitis, 463 of 1038 (44.6%) patients had acute seizures. The initial seizure types were categorized into focal seizures (n = 125/463, 27%), generalized tonic-clonic seizures (n = 149/463, 32.2%), myoclonic seizures (n = 56/463, 12.1%), and secondarily generalized seizures (n = 133/463, 28.7%). The frequency of seizures was classified into single seizure (n = 83/463, 17.9%), repetitive seizures (n = 227/463, 49%), status epilepticus (n = 100/463, 21.6%), and refractory status epilepticus (n = 53/463, 11.4%).

### Antiepileptic drugs for encephalitis-related acute symptomatic seizures

There were 463 patients with acute symptomatic seizures in the acute phase of encephalitis, including 330 patients who received antiepileptic drugs after admission. Of patients with single seizure (n = 83), 53 did not receive any antiepileptic drugs and 30 received antiepileptic drugs. All responded to diazepam or lorazepam. For patients with repetitive seizures (n = 227), 147 were treated with antiepileptic drugs. As regards initial antiepileptic drugs, 17 of 147 patients (11.6%) were controlled after diazepam or lorazepam, while 130 patients were treated with second-line antiepileptic drugs. Sixteen patients were initially treated with oral antiepileptic drugs as second-line for seizure control, including oral carbamazepine (n = 10), clonazepam (n = 3), lamotrigine (n = 1), carbamazepine plus clonazepam (n = 1), and carbamazepine plus lamotrigine (n = 1). The remaining 114 patients received intravenous antiepileptic drugs as second-line treatment.

Of the 114 patients, 96 (84.2%) responded to the second-line drugs. Phenytoin was administered to 61 patients and phenobarbital was administered to 51 patients, which resulted in the cessation of clinical seizures in 46 (75.4%) and 48 (94.1%) of patients, respectively. Phenobarbital was more effective than phenytoin (*p* = 0.009). In all of the refractory patients, phenobarbital and valproic acid were effective as a third choice of drug. However, there were no statistically significant differences between the types of drugs. The order and effectiveness of each intravenous antiepileptic drug used were summarized in Table 2.

**Table 2:** Sequence and efficacy of intravenous antiepileptic drugs in patients with acute symptomatic seizures after pediatric encephalitis (n = 330).

Treatment */*, (*%)	1st	2nd	3rd	4th	5th	6th	7th	8th	Cumulative efficacy
**Single seizure (n = 30)**									
Benzodiazepines	30/30 (100%)								30/30 (100%)
**Repetitive seizures (n = 147)**									
Benzodiazepines	17/147 (11.6%)								17/147 (11.6%)
Phenytoin		46/61 (75.4%)							46/61 (75.4%)
Phenobarbital		48/51 (94.1%)	9/9 (100%)						57/60 (95%)
Valproic acid		2/2 (100%)	1/1 (100%)						3/3 (100%)
Total	47/177 (26.6%)	96/114 (84.2%)^a^	10/10 (100%)^b^						
**Status epilepticus or refractory status epilepticus(n = 153)**									
Benzodiazepines	0/153 (0%)								
Phenytoin		49/104 (47.1%)	6/17 (35.3%)						55/121 (45.4%)
Phenobarbital		25/49 (51%)	13/47 (27.6%)						38/96 (39.5%)
Valproic acid			5/13 (38.5%)	4/23 (17.4%)	4/7 (57.1%)				13/43 (30.2%)
Midazolam CIV			1/2 (50%)	15/24 (62.5%)	0/16 (0%)				16/42 (38.1%)
Propofol CIV				1/1 (100%)	3/3 (100%)	2/4 (50%)			6/8 (75%)
Thiopental CIV				2/2 (100%)		1/1 (100%)	0/1 (0%)	1/1 (100%)	4/5 (80%)
TPM+Lidocaine						7/11 (63.6%)	0/2 (0%)		7/13 (53.8%)
TPM+HD-PB					2/2 (100%)	1/1 (100%)	2/3 (66.7%)	0/2 (0%)	5/8 (62.5%)
Total	0/153 (0%)	74/153 (48.4%)	25/79 (31.6%)^c^	22/50 (44%)	9/28 (32.1%)	11/17 (64.7%)^d^	2/6 (33.3%)^e^	1/3 (33.3%)	

*/*: number of seizure termination/number of trial

(*):(efficacy, %)

Benzodiazepines: diazepam or lorazepam

CIV: continuous intravenous administration; TPM: high-dose topiramate; HD-PB: high-dose phenobarbital

Seizures were not clearly terminated but gradually subsided after treatment with other oral antiepileptic drugs in (a) 16 patients, (b) 8 patients, (c) 4 patients, (d) 1 patient, and (e) 1 patient.

All of the patients with status epilepticus and refractory status epilepticus (n = 153) were treated with intravenous antiepileptic drugs after initial diazepam or lorazepam. Seventy-four (48.4%) of the 153 patients were controlled after a second antiepileptic drug. Phenytoin was administered to 104 patients and phenobarbital was administered to 49 patients, which resulted in the cessation of clinical seizures in 49 (47.1%) and 25 (51%) of the patients, respectively. Phenobarbital was more effective than phenytoin, but not statistically significant (*p* = 0.056).

In the refractory patients, phenytoin was effective as a third choice in six of 17 patients (35.3%), phenobarbital was effective in 13 of 47 patients (27.6%), valproic acid was effective in five of 13 patients (38.5%), and midazolam was more effective in one of two patients (50%). However, there were no statistically significant differences among the types of drug. The sequence and effectiveness of each of the intravenous antiepileptic drugs used were summarized in Table 2.

Fifty-three patients did not respond to any of the third-line drugs and developed refractory status epilepticus. Seizures were stopped by multiple high-dose suppressive therapy in 45 children, including thiopental (n = 4/5, 80%), propofol (n = 6/8, 75%), high-dose topiramate combined with high-dose phenobarbital (n = 5/8, 62.5%), high-dose topiramate combined with high-dose lidocaine (n = 7/13, 53.8%), midazolam (n = 15/40, 37.5%), and valproic acid (n = 8/30, 26.7%). However, the relative risk for respiratory depression and hypotension was higher in the use of thiopental or propofol than in high-dose topiramate combined with high-dose lidocaine or high-dose phenobarbital for seizure control (10/13 vs. 12/21, *p* = 0.030).

All of the patients in this study who developed respiratory depression required ventilatory support. Six of eight (75%) patients who used thiopental and five (100%) who used propofol developed hypotension. Six of 13 (46.2%) who used high-dose topiramate combined with high-dose lidocaine and three of eight (37.5%) patients who used high-dose phenobarbital also developed hypotension. Seizures gradually subsided after a ketogenic diet in three patients. Five of 53 (9.4%) patients did not respond to any antiepileptic drugs.

### Mortality and outcomes

Fifteen patients were discharged against medical advice while 33 died during their hospital stay due to uncal herniation (n = 16), sepsis (n = 11), thiopental- or propofol-induced profound hypotension (n = 5), and ventricular fibrillation/ventricular tachycardia (n = 1). Mortality was related to poor control of status epilepticus. Fourteen of 53 (26.4%) patients with refractory status epilepticus died, but only 19 of 971 (2.0%) with non-refractory status epilepticus died (*p*<0.001).

### Antiepileptic drugs and postencephalitic epilepsy at one year of follow-up

**Fig 1:**
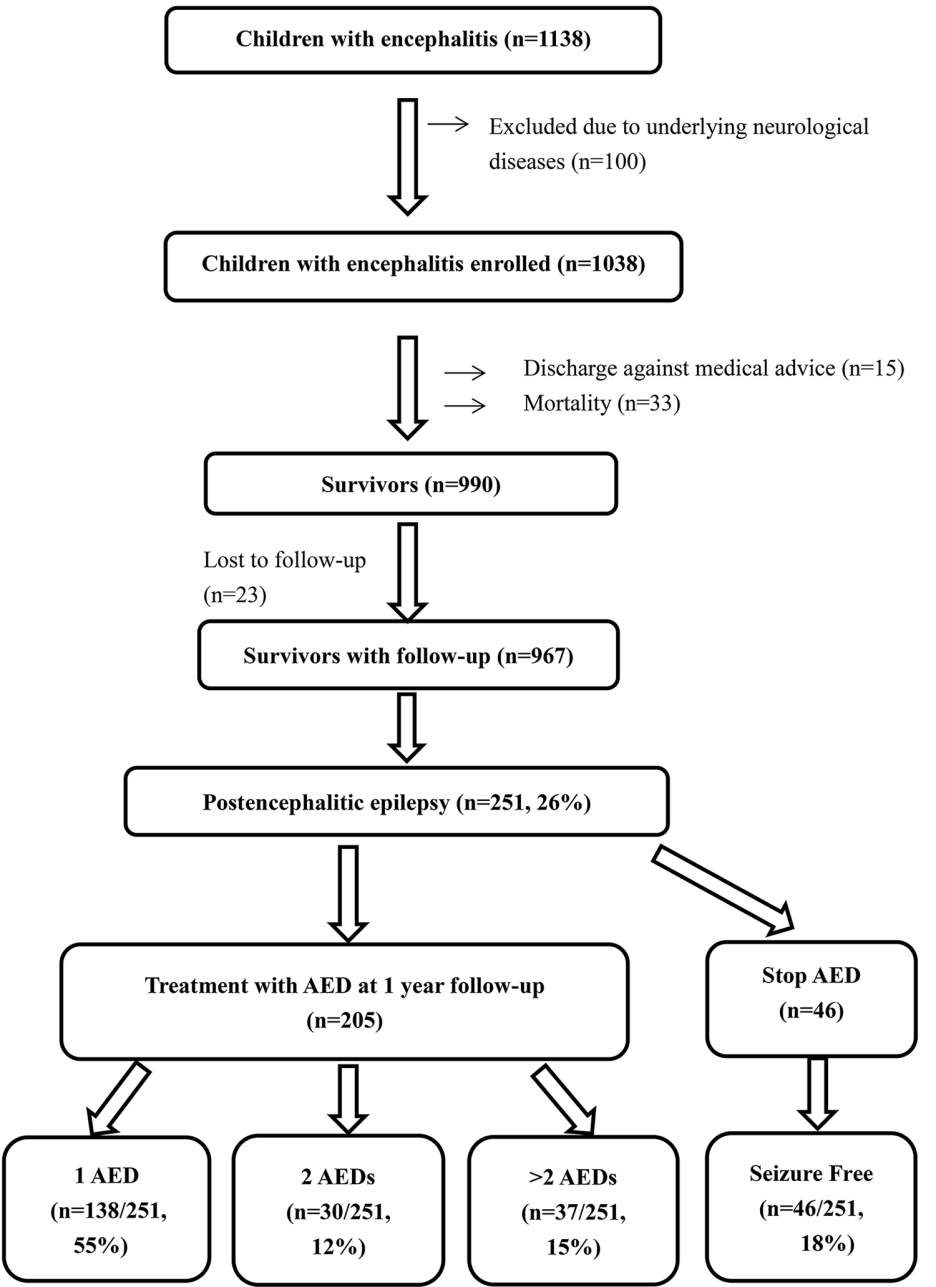
Flow diagram of children with postencephalitic epilepsy.

A total of 990 survivors were discharged, including 268 (27.1%) who were discharged with antiepileptic drug treatment (Fig 1). Excluding 23 patients who were lost to follow-up and excluded from the analysis, 251 of the 967 (26%) patients developed postencephalitic epilepsy. Of the 251, 46 (18.3%) successfully stopped taking antiepileptic drugs within one year, while 205 (78.7%) continued to receive antiepileptic drugs. Among them, 138 (54.9%) received a single antiepileptic drug for seizure control, 30 (11.9%) received two antiepileptic drugs, and 37 (14.7%) received more than two antiepileptic drugs. Phenobarbital and clonazepam were the most common drugs used in combination therapy for postencephalitic epilepsy. Of the 251 patients, 214 (85.3%) had favorable outcomes and 37 (14.7%) had intractable seizures. The antiepileptic drugs used for postencephalitic epilepsy were summarized in Table 3.

**Table 3:** Choice of antiepileptic drugs for postencephalitic epilepsy in 205 children.

AED	Number (%)	PB	BZD	VPA	CBZ	TPM	OXC	LEV	PHT	LA	VGB	LTG	AZA	GBP
Single AED	138 (67.3%)	72	16	13	19	7	3		5	2		1		
Multiple AEDs														
2 AEDs	30 (14.6%)	15	12	10	8	6	2		2	3		1		1
3 AEDs	24 (11.7%)	10	11	11	7	11	3	7	2	2	5	3		
4 AEDs	8 (3.9%)	6	6	4	1	3	1	2			4	1		
5 AEDs	4 (2.0%)	4	4	3	1	3	2	1		1			1	
6 AEDs	1 (0.5%)	1			1	1				1		1	1	
Total	205 (100%)	108	49	41	37	31	11	10	9	9	9	7	2	1

AED: antiepileptic drugs; PB: phenobarbital; BZD: benzodiazepines (diazepam or lorazepam); VPA: valproic acid; CBZ: carbamazepine; TPM: topiramate; OXC: oxcarbazepine; LEV: levetiracetam; PHT: phenytoin; LA: phenytoin+phenobarbital; VGB: vigabatrin; LTG: lamotrigine; AZA: acetazolamide; GBP: gabapentin

A total of 990 survivors were enrolled, including 268 (27.1%) who were discharged with antiepileptic drugs (AEDs) treatment. Thirty-seven children (37/251, 15%) with intractable epilepsy received more than two AEDs at one year of follow-up.

## Discussion

Central nervous system infections are a known risk factor for the development of acute symptomatic seizures and represent a major risk factor for acquired epilepsy [20]. In a recent study of acute seizures in central nervous system infections, 23% of patients have acute seizures and 73% have encephalitis, which is a significantly more frequent etiology than meningitis. Viral encephalitis has been shown to result in a 14-fold increase in the risk of acute seizures [21]. In the present study, 463 (44.6%) children with encephalitis have had seizures in the acute phase, suggesting that encephalitis leads to a high percentage of the presentation of acute seizures, especially in pediatric encephalitis. In this cohort, 153 (33%) patients with acute seizures have status epilepticus, including 53 (34.6%) who developed refractory status epilepticus. Thus, once seizures develop in the acute phase of pediatric encephalitis, clinicians should be aware of the possible development of refractory status epilepticus.

Encephalitis is most often evidenced in first episode of status epilepticus and appears to be a marker for morbidity and mortality [8]. In the present study, 14/53 (26.4%) of patients with refractory status epilepticus died, whereas only 19/971 (2.0%) of patients with non-refractory status epilepticus died (*p*<0.001). Therefore, mortality is significantly related to the presence of refractory status epilepticus in the pediatric patients with encephalitis. The underlying cause is well known to be the influential factor on the outcome of status epilepticus [22,23]. Therefore, clinicians should do their best to treat the underlying disease and provide effective antiepileptic drugs for the best possible outcomes.

In general, seizures are initially treated with boluses of intravenous lorazepam or diazepam as first-line drugs, followed by intravenous infusions of phenytoin and/or phenobarbital as second-line drugs. Phenytoin is reported to be ineffective for febrile status epilepticus [24,25]. Sugai suggests that pentobarbital is effective for prolonged status epilepticus whereas phenytoin is for cluster convulsive status epilepticus [26]. In this study, phenobarbital (97.5%) is more effective than phenytoin (75.4%) for controlling repetitive seizures (*p* = 0.009). Phenobarbital may therefore be a suitable second-line antiepileptic drug for seizures in pediatric encephalitis.

In the current study, 53 patients did not respond to the initial antiepileptic drug and developed refractory status epilepticus. They responded to high-dose suppressive therapy that included intravenous thiopental (80%), propofol (75%), high-dose topiramate combined with high-dose phenobarbital (62.5%), or high-dose lidocaine (53.8%). In addition, three patients responded to a ketogenic diet combined with antiepileptic drugs. In terms of adverse effects, 5 of 13 (38.5%) patients died due to thiopental- (n = 4) or propofol-induced (n = 1) profound hypotension. Therefore, we suggest that high-dose topiramate combined with intravenous high-dose phenobarbital or high-dose lidocaine may be considered as an alternative third-line treatment for refractory status epilepticus due to encephalitis [19,27,28]. A ketogenic diet may also be considered [29–31].

Regarding the long-term management of postencephalitic epilepsy, it is reported that high-dose phenobarbital is the most effective agent for seizure control during the recovery phase and chronic phase of acute encephalitis with refractory repetitive partial seizures [26,27]. In the current study (n = 205, 1 year follow-up), 168 (82%) patients with postencephalitic epilepsy have favorable outcomes and 37 (18%) have intractable epilepsy. Phenobarbital and clonazepam are the most common drugs used alone or in combination for postencephalitic epilepsy, followed by valproic acid and carbamazepine. The role of new antiepileptic drugs in postencephalitic epilepsy warrants further research.

The present study has some limitations. First, there was no routine electroencephalogram monitoring for every patient. Thus, the subgroup of non-convulsive status epilepticus is not identified. Second, bias may exist in the selection of antiepileptic drugs because this is a retrospective study. Third, the definitions of acute symptomatic seizure and postencephalitic epilepsy are modified from those proposed by the International League Against Epilepsy Epidemiology Commission. Fourth, this is not a population-based study. Further prospective studies are necessary.

## Conclusions

Children with encephalitis may have a high rate (26%) of postencephalitic epilepsy, 15% of them having intractable epilepsy. The mortality rate is significantly high when they develop refractory status epilepticus. Based on the findings here, the recommended treatment for acute seizures or status epilepticus in pediatric encephalitis should include several considerations. First, phenobarbital is preferred over phenytoin as a second-line agent in treatment. Second, high-dose topiramate combined with intravenous high-dose phenobarbital or high-dose lidocaine may be considered third-line treatment for refractory status epilepticus.

## Supporting Information

S1 File The demographic data of 1038 encephalitic children. During the study period, 1138 patients were discharged with a diagnosis of acute encephalitis. After excluding 100 patients with underlying neurologic diseases, 1038 previously healthy patients diagnosed with acute encephalitis were enrolled in the study.
(XLS)
